# A Whole-Transcriptome Approach to Evaluating Reference Genes for Quantitative Gene Expression Studies: A Case Study in *Mimulus*

**DOI:** 10.1534/g3.116.038075

**Published:** 2017-03-03

**Authors:** Kimmy A. Stanton, Patrick P. Edger, Joshua R. Puzey, Taliesin Kinser, Philip Cheng, Daniel M. Vernon, Nancy R. Forsthoefel, Arielle M. Cooley

**Affiliations:** * Department of Biology, Whitman College, Walla Walla, Washington 99362; † Department of Horticulture, Michigan State University, East Lansing, Michigan 48824; ‡ College of William and Mary, Williamsburg, Virginia 23185

**Keywords:** expression stability, *Mimulus guttatus*, *Mimulus luteus*, quantitative RT-PCR, RNA-seq

## Abstract

While quantitative PCR (qPCR) is widely recognized as being among the most accurate methods for quantifying gene expression, it is highly dependent on the use of reliable, stably expressed reference genes. With the increased availability of high-throughput methods for measuring gene expression, whole-transcriptome approaches may be increasingly utilized for reference gene selection and validation. In this study, RNA-seq was used to identify a set of novel qPCR reference genes and evaluate a panel of traditional “housekeeping” reference genes in two species of the evolutionary model plant genus *Mimulus*. More broadly, the methods proposed in this study can be used to harness the power of transcriptomes to identify appropriate reference genes for qPCR in any study organism, including emerging and nonmodel systems. We find that RNA-seq accurately estimates gene expression means in comparison to qPCR, and that expression means are robust to moderate environmental and genetic variation. However, measures of expression variability were only in agreement with qPCR for samples obtained from a shared environment. This result, along with transcriptome-wide comparisons, suggests that environmental changes have greater impacts on expression variability than on expression means. We discuss how this issue can be addressed through experimental design, and suggest that the ever-expanding pool of published transcriptomes represents a rich and low-cost resource for developing better reference genes for qPCR.

qPCR is the premier method for quantifying gene expression because of its simplicity, accuracy, and low cost. However, the quantification accuracy of qPCR is dependent on normalization against reference genes to reduce the impact of technical noise and variation in sample preparation. qPCR data normalization is crucial for the reliable quantification of expression levels, so care must be taken to choose a reliable reference gene that has low variation in expression across diverse sample types (Dheda *et al.* 2005; Gutierrez *et al.* 2008). Traditionally, high expression housekeeping genes involved in basic cellular functions were used for qPCR normalization based on the assumption that they would be stably expressed (Thellin *et al.* 1999). Unfortunately, these traditional housekeeping reference genes, such as ubiquitin-conjugating enzyme (UBC), polyubiquitin (UBQ), β-actin, α- and β-tubulin, and glyceraldehyde 3-phosphate dehydrogenase (GAPDH), can exhibit surprisingly high expression variance in some species, or among different environmental conditions (Brunner *et al.* 2004; Czechowski *et al.* 2005; Dheda *et al.* 2004; Suzuki *et al.* 2000).

In efforts to find alternatives to housekeeping genes, high-throughput technologies have been used to survey whole transcriptomes for novel, stably expressed genes. Microarrays have been successfully used for novel reference gene identification in a variety of plants, including *Arabidopsis thaliana*, *Eucalyptus*, and soybean (Czechowski *et al.* 2005; Libault *et al.* 2008; de Oliveira *et al.* 2012). However, RNA-seq, a potentially more effective high-throughput method, has rarely been employed. RNA-seq has many advantages over microarrays: it does not require an assembled genome (Haas and Zody 2010; Grabherr *et al.* 2011; Robertson *et al.* 2010), it has the power to identify novel transcripts and splice variants (Trapnell *et al.* 2010), and it is sensitive enough to quantify transcripts with very low expression levels (Marioni *et al.* 2008). In addition, RNA-seq is fast, relatively inexpensive, and shows minimal variation across technical replicates (Marioni *et al.* 2008; Wang *et al.* 2009; Mortazavi *et al.* 2008; Nagalakshmi *et al.* 2008). For all of these reasons, RNA-seq is an attractive, whole-transcriptome method for the detection of stably expressed genes and the identification of novel reference genes for qPCR normalization. This approach has rarely been used to evaluate potential qPCR reference genes [but see Chang *et al.* (2012), Yang *et al.* (2014), and Zhuang *et al.* (2015)].

A potential pitfall of both the microarray and the RNA-seq approach to reference gene selection is that there are no accepted practices for the analysis of expression variability within whole transcriptomes. Many methods for analyzing expression variability from qPCR data have been developed, including geNorm, BestKeeper, and NormFinder (Andersen *et al.* 2004; Vandesompele *et al.* 2002; Pfaffl *et al.* 2004), but these programs can only analyze the expression data from a handful of genes at a time and, thus, are not useful for exploring whole transcriptomes. Without an established method for analysis, many diverse methods have been adopted for estimating expression variability within whole transcriptomes, including coefficient of variation (CV) calculations (Czechowski *et al.* 2005), fold change cut-offs (Yang *et al.* 2014), and *P*-value cut-offs (Libault *et al.* 2008). However, no comparison of the different methods is currently available; each of the earlier studies included only a single whole-transcriptome measure of expression variability.

One system in which a transcriptomic approach to reference gene selection has great potential to advance gene expression studies is the monkeyflower genus *Mimulus* [recently split into genera *Mimulus* and *Erythranthe* (Barker *et al.* 2012)]. *Mimulus* has become a widely used model for evolutionary genetic studies because of its phenotypic, ecological, and genetic variation, with centers of species diversity in both North and South America (Wu *et al.* 2008; Sobel and Streisfeld 2013; Beardsley and Olmstead 2002; Twyford *et al.* 2015). *Mimulus* is a powerful system for genetic studies due to the interfertility of diverse species and the availability of genomic resources, including the genome sequence of *Mimulus guttatus*, *M. cardinalis*, *M. lewisii*, and *M. luteus* (Hellsten *et al.* 2013; Yuan *et al.* 2013; Edger *et al.* 2016). Yet, despite the utility of *Mimulus* for studying the evolution of genes and gene expression, the only evaluation of qPCR reference genes to date is a nonquantitative assessment of six housekeeping genes (Scoville *et al.* 2011). A rigorous and quantitative genome-wide analysis of candidate qPCR reference genes is therefore of special utility for advancing evolutionary genetic studies in *Mimulus*.

In this study, we systematically and quantitatively evaluate a panel of traditional reference genes and screen whole transcriptomes to identify a set of novel reference genes that can be used for qPCR expression studies in *Mimulus*. We utilize whole-transcriptome RNA-seq libraries from two species: *M. guttatus*, a North American diploid, and *M. luteus* var. *luteus*, a Chilean allotetraploid (Mukherjee and Vickery 1962; Vallejo‐Marín *et al.* 2015). We further develop the toolkit for transcriptome-enabled reference gene selection by comparing the utility of two distinct methods—the “CV method” and the “fold change cut-off method”—for identifying novel stably expressed genes from RNA-seq data.

In these two *Mimulus* species, which differ in their ecology, ploidy, and level of resource development, we find that both the CV and fold change methods identify a similar set of novel reference genes. We propose that these highly stable genes provide a good starting pool of candidate reference genes for qPCR expression studies in *Mimulus*, and report that some traditional reference genes are also satisfactory according to standard quantitative guidelines for qPCR. In addition, we propose a workflow that incorporates either the CV or the fold change method to screen whole transcriptomes for novel reference genes in other systems. Across environmentally and genetically different plants, we found that gene expression means were relatively similar but expression variability fluctuated dramatically. Based on this finding, we suggest that transcriptomes should either be specific to the samples used for the planned qPCR study or should cover a wide span of biological and environmental diversity, in order for reference genes to be selected with high confidence.

## Materials and Methods

### Plant materials

**Figure 1:**
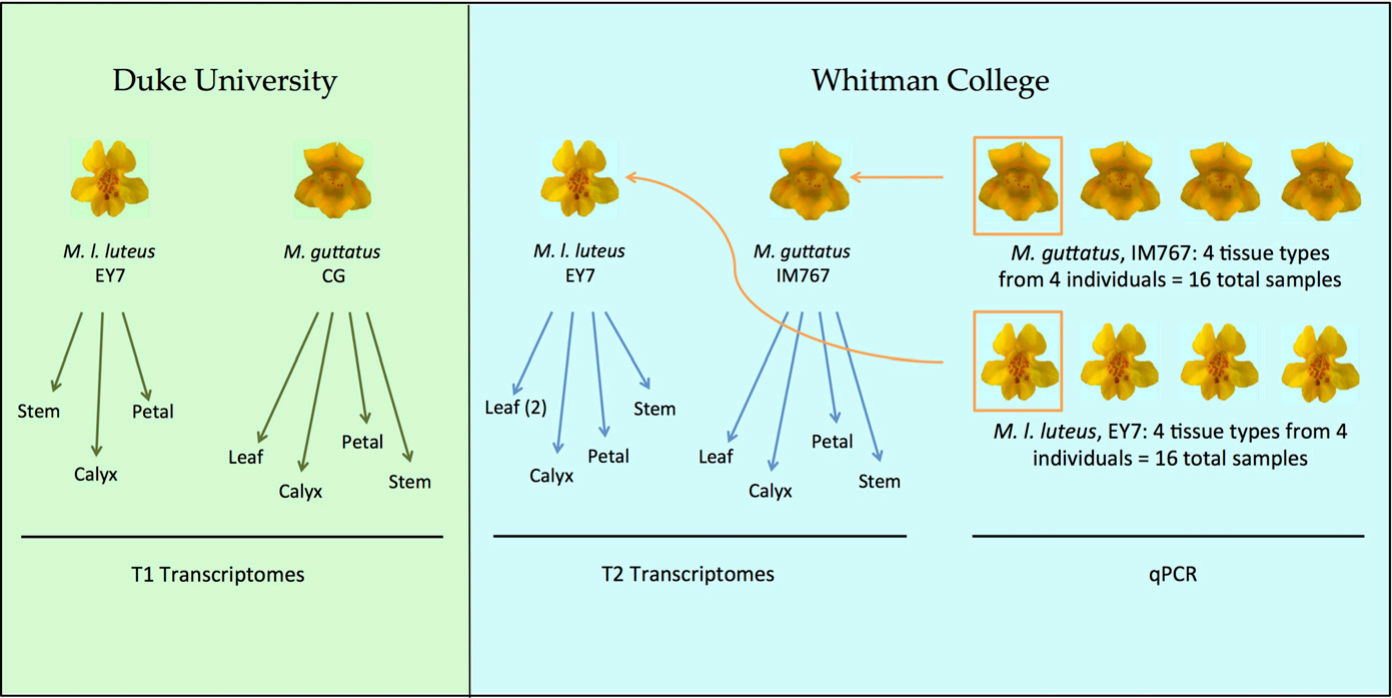
Sources of the plant materials that provided RNA for RNA-seq and qPCR. Because no leaf sample was available for *M. l. luteus* T1, two leaves were collected and sequenced for *M. l. luteus* T2, to enable leaf-to-leaf comparisons within *M. l. luteus. M. guttatus* genotype CG was collected in Dublane, Scotland; *M. l. luteus* highly inbred line EY7 was originally collected from El Yeso, Chile (Cooley *et al.* 2008); *M. guttatus* highly inbred line IM767 was originally collected from Iron Mountain, OR (Willis 1999). qPCR, quantitative polymerase chain reaction; RNA-seq, RNA sequencing.

Two batches of each species were grown in separate greenhouses, providing the RNA samples for both RNA-seq and qPCR (Figure 1). *M. guttatus* genotype CG (Dublane, Scotland) and *M*. *luteus* var. *luteus* inbred line EY7 [El Yeso, Chile, see Cooley *et al.* (2008)]were grown at Duke University (NC). Transcriptome T1 was produced for *M. l. luteus* using stem, calyx, and petal tissues from a single individual grown at Duke University (NC). For *M. guttatus*, stem, calyx, petal, and leaf were sampled from two CG plants grown at Duke University. For transcriptome T2, *M. guttatus* inbred line IM767 [Iron Mountain, OR, see Willis (1999)] and *M. l. luteus* inbred line EY7 (El Yeso, Chile) were grown at Whitman College (WA). RNA from four tissue types of a single individual of each species grown at Whitman College was sequenced to produce the second set of transcriptomes (T2). Two different leaf samples were collected from *M. l. luteus* and processed separately (L2A and L2B), to compensate for the lack of a leaf transcriptome in T1. RNA from four tissue types of four individuals (one of which was the same individual used for the T2 transcriptomes) from each species grown at Whitman College was extracted for use in qPCR.

In the Whitman greenhouse, plants were grown with supplemental 14 hr lighting in Miracle-Gro potting soil (N:P:K = 0.21:0.11:0.16, The Scotts Company, Marysville, OH). Plants were maintained on “self-watering” capillary action flats with once-daily top-watering. Greenhouse temperatures, as recorded by a wall sensor, ranged from 16° to 36° daily. Plants were fertilized twice weekly with Miracle-Gro Bloom Booster (N:P:K = 15:30:15). In the Duke greenhouse, plants were grown with supplemental 16 hr lighting with twice-daily watering. Greenhouse temperatures ranged from 12° to 21° daily. Plants were fertilized with Peter’s Professional fertilizer every 2 wk, alternating between general purpose (N:P:K = 20:10:20) and low-phosphorus (N:P:K = 15:0:15) formulas, and fertilized with Jack’s Classic Blossom Booster (JR Peters INC, PA) (N:P:K = 10:30:20) every week to enhance flowering.

Tissue was harvested from young, budding plants, usually between the first and third flower. Four tissue types were collected: young leaf (<2.5 cm) near apical and lateral meristems, whole calyx from unemerged buds, petal (with stamen and pistil removed) from unemerged buds, and stem (∼2.5 cm segments) from newer plant growth. Tissue samples were flash frozen in liquid nitrogen and stored at −80° until the date of RNA extraction.

### Transcriptome preparation and assembly

Total RNA was isolated from four tissue types (stem, leaf, calyx, and petal) from both *M. guttatus* and *M. l. luteus*. At Whitman, the Agilent Plant RNA Isolation Kit (Santa Clara, CA) was used, and at Duke, the Zymo Research Direct-Zol RNA MiniPrep (Irvine, CA) was used, following the manufacturer’s protocol, with on-column DNase I and elution in nuclease free water heated to 65°. RNA concentration and integrity were assessed using a NanoDrop Lite spectrophotometer (Thermo Fisher Scientific, DE) or Qubit fluorometer (Thermo Fisher Scientific).

Whole-transcriptome, RNA-seq libraries were constructed for four tissue types from each of two biological replicates (T1 and T2) for both *M. guttatus* and *M. l. luteus* (see Figure 1). T1 transcriptomes were prepared using the TruSeq RNA kit (Illumina, San Diego, CA) and then sequenced with single-end 100 bp reads using one lane of an Illumina HiSeq-2000 at the University of Missouri DNA core. T2 transcriptomes were prepared using the Kapa Stranded mRNA-Seq kit (Kapa Biosystems, Wilmington, MA) and were sequenced using one lane of an Illumina Hiseq-2500 at the Duke University DNA core.

All Illumina reads were quality filtered using NextGENe v2.3.3.1 (SoftGenetics, State College, PA). Adapter sequences and reads with a median quality score of <22 were removed, reads were trimmed at positions that had three consecutive bases with a quality score of <20, and any trimmed reads with a total length <40 bp were removed. This resulted in ∼87.9% of the reads passing the quality-score filter. Expression levels, in FPKM (fragments per kilobase per million reads), were determined for a total of 25,465 genes in *M. guttatus* (diploid) and 46,855 genes in *M. l. luteus* (tetraploid). Quality-filtered reads for each library were aligned to the respective genomes using NextGENe v2.3.3.1. Only uniquely mapped reads were counted, using the following parameters: A. matching Requirement: >40 Bases and >99%, B. Allow Ambiguous Mapping: FALSE, and C. Rigorous Alignment: TRUE. This resulted in the alignment of over 74.4 million reads to the diploid *M. guttatus* genome and 107.4 million reads to the tetraploid *M. l. luteus* genome.

Genome completeness of the allotetraploid *M. l. luteus* in terms of gene content was assessed using BUSCO (Simão *et al.* 2015) with the default setting and a set of universal single-copy orthologs. The vast majority of BUSCO groups, 931 of 956 (97.4%), were identified in the *M. l. luteus* genome assembly, and 837 of those had duplicates. The high percentage of duplicate genes in this analysis indicates that homeologs were not collapsed during the assembly of the genome. This is further supported by comparative genomic analyses of both *Mimulus* genomes (Edger *et al.* 2016), revealing a 2:1 genome-wide ratio of *M. l. luteus* (tetraploid): *M. guttatus* (diploid) syntenic blocks.

### Analysis of RNA-seq libraries

Within each species, genes with expression levels lower than five FPKM in any of the eight transcriptomes were excluded from any of the further stability analyses. We reasoned that such low-expression genes would make poor qPCR references due to the difficulties in detecting and quantifying their expression. After their removal, a total of 7225 genes in *M. guttatus* and 10,755 genes in *M. l. luteus* were evaluated. Two methods were used for the analysis of expression stability: simple CV calculations and exclusion of differentially expressed genes [fold change method (Robinson *et al.* 2010)].

#### For the CV method:

Calculations for mean expression (mean), SD, and the CV were executed in Microsoft Excel or in R (Pumpkin Helmet, v.3.1.2). CV was calculated as SD/mean. Mean and SD were measured over the four tissue types of both biological replicates (eight samples in total) for each species. We adopted a CV cut-off for stable genes of 0.5, which was the cut-off for stable expression across heterogeneous samples advocated by Hellemans *et al.* (2007).

#### For the fold change method:

Log fold change was used to evaluate differential expression in pairwise sample comparisons. Genes with a high fold change (>0.4 in *M. guttatus* and 0.3 in *M. l. luteus*) in any pairwise sample comparison were eliminated until a final list of stably expressed genes was obtained (Supplemental Material, Table S1). The cut-off values used in this study were selected so as to obtain a short list of genes with low variation in expression; the appropriate cut-off value can vary depending on the samples being analyzed and the overall goal of the analysis. The edgeR program (v. 3.12.0) was used to calculate log fold change because the program normalizes expression values by library size for each sample, but any method of fold change calculation can be used. The edgeR program was accessed through Bioconductor and analysis was executed in R.

### Gene annotation

Stably expressed genes were annotated based on the agreement between BLAST results from the NCBI nucleotide database (http://blast.ncbi.nlm.nih.gov/) and from the annotated *M. guttatus* v.2 genome in the Phytozome v.10 database (http://phytozome.jgi.doe.gov/). Traditional reference genes were identified in the RNA-seq datasets in a three-part method. First, known *A. thaliana* sequences for traditional reference genes 60s ribosomal protein L8 (L8), actin 2/7 (ACT), actin 11 (ACT1), β-tubulin 2 (TUB), ubiquitin 5 (UBQ), UBC 25, peroxin 4 (PEX), GAPDH-C1 (GAP), and EF1-α (EF1) (see Table S2) were used in a BLAST search against the *M. guttatus* v.2 genome in the Phytozome v.10 database in order to identify the appropriate *M. guttatus* homologs. Once a gene match with the correct annotation was identified in Phytozome, a short (∼20 bp) sequence from the coding region was then used to identify transcripts from the *M. guttatus* and *M. l. luteus* RNA-seq libraries. The resulting *M. guttatus* and *M. l. luteus* transcripts were used in a BLAST search against the NCBI nucleotide database to ensure that they had been correctly identified.

### qPCR genes

Eight genes were selected for validation via qPCR (Table S3). Four traditional reference genes were selected based on both their widespread use in qPCR reference gene literature and on the ease of designing copy-specific primers. The four traditional genes chosen were ACT, GAP, PEX, and UBC. See the above section on *Gene annotation* for methods of gene identification within the transcriptome. Four additional genes were chosen based on their apparent stability across T1 tissues in both species, but were later found to be unstably expressed across T2 tissues (see Table S3). However, these genes were retained for analysis in order to compare the qPCR and RNA-seq methods. The four genes chosen were mediator of RNA polymerase 12 (MRP), pectin acetylesterase (PAE), receptor-like kinase (RPK), and FYVE zinc-finger transcription factor (ZNF). The *M. guttatus* GenBank accession numbers for these eight genes, cataloged under *Erythranthe guttata* (Barker *et al.* 2012), are: ACT = XM_012974510.1, GAP = XM_012999102.1, PEX = XM_013002418.1, UBC = XM_012995233.1, MRP = XM_012984744.1, PAE = XM_012984356.1, RPK = XM_012985914.1, and ZNF = XM_013000433.1.

### qPCR primer design

qPCR primers were designed using Primer3 (http://biotools.umassmed.edu/bioapps/primer3_www.cgi) with the following criteria: *T*_m_ of 60 ± 3°, PCR amplicon length of 130–250 bp, primer length of 18–25 bp, and GC content of 35–60%. The *T*_m_ criterion was relaxed for UBC to 55 ± 3° to enable the discovery of suitable primers. Primers were designed to optimally sit as close to the 3′-end of the transcript as possible and to span an intron, but these criteria were relaxed in an effort to design primers that are homeolog-specific in the allotetraploid *M. l. luteus*. *M. l. luteus* primers were aligned with BLAST against the *M. l. luteus* (Illumina masked v1.1) genome in CoGe (https://genomevolution.org/CoGe/) to ensure homeolog and paralog specificity. *M. guttatus* primers were aligned with BLAST against the *M. guttatus* genome (JGI hardmasked vV2) in CoGe to ensure copy specificity. Primers were synthesized by Invitrogen (Life Technologies). See Table S4 for the full list of primer pairs.

To verify primer specificity, PCR products were amplified by Taq DNA polymerase in a Mastercycler Nexus (Eppendorf, Germany), gel purified using the E.Z.N.A. kit (Omega Biotek), and Sanger sequenced by Eton Bioscience. Although all primers produced a single band on an agarose gel, the gel extraction step was included to produce cleaner and more concentrated sequencing products. Sequencing confirmed the copy specificity of all primer pairs except for the *M. l. luteus* RPK and PEX primer pairs, which targeted two and three paralogs, respectively.

### cDNA synthesis

cDNA was synthesized from 1 μg of total RNA and a mixture of oligo dT and random primers using the Quanta qScript cDNA Synthesis kit (Quanta BioSciences, MD) and following the manufacturer’s protocol. cDNA was stored at 4° and unused RNA was stored at −80°.

Quality controls for cDNA were twofold. First, all RNAs and cDNAs were checked for the absence of genomic DNA contamination using primers that surround an actin intron (5′-CCCAAGGCTAACAGGGAGAA-3′ and 5′-GTGCTGGATTCTGGTGACG-3′). Second, gene expression estimates were obtained from the 3′- *vs.* 5′-ends of a single gene. A 3′/5′ ratio substantially greater or less than 1 may indicate degradation of the mRNA template, or incomplete processivity of the reverse transcription reaction. The MIQE guidelines (Bustin *et al.* 2009) suggest a range of 0.2–5.0 for samples to be used in qPCR. The 3′/5′ ratio of the receptor-like protein kinase cDNA was tested for all tissue types in each individual used in this study, using two primer pairs that amplify in the 5′ region (5′-TGGGCTCGAGTATTTTGCTT-3′ and 5′-TGCTTCCTAATCCAAAGATACCA-3′) or the 3′ region (5′-CCTGAGGGTGACAAGACACA-3′ and 5′-ATCAATGGACAAAAGCAGGC-3′) ∼1 kb away from each other. Some 3′/5′ ratios were found to be >5 (see Table S5). This could result in an underestimation of expression for genes with primers in the 5′ region of the gene, which includes ACT in both species and the *M. guttatus* ZNF. The 3′/5′ ratios also had a tissue bias, with all stem cDNA samples and some of the calyx cDNA samples having values >5.

### qPCR conditions

Comparative qPCR was performed for four biological replicates (all from plants grown at Whitman College, see *Plant materials*) and three technical replicates for each tissue type (leaf, stem, petal, and calyx) from each of the two species (*M. guttatus* and *M. l. luteus*). A total of eight genes were selected for qPCR validation (see section *qPCR genes* and Table S3) using the primers listed in Table S4. Reactions contained 1 × SYBR Green Master Mix (Brilliant III Ultra-Fast SYBR Kit, Agilent Technologies, CA), 400 nM of primer (except for when amplifying PEX4 from *M. l. luteus*, where 500 nM of primer was used), 1 μl of 1:500 diluted ROX dye, and 1 μl of cDNA (50 ng/μl), in a final volume of 12.5 μl. PCR reactions were performed in either optical eight-well PCR strips (Agilent Technologies) or optical 96-well plates (Greiner Bio-One, Belgium) using the Stratagene Mx3000P qPCR system (Agilent Technologies). Samples were amplified for 40 cycles of 10 sec at 95° and 20 sec at the appropriate annealing temperature (see Table S4), after an initial denaturation step at 95° for 3 min. An additional dissociation curve was recorded after cycle 40 by heating from 55° to 95° with a ramp speed of 0.01° per second (Figure S1). Raw qPCR fluorescence data were collected and analyzed by the default settings of the MxPro software v.4.10 (Agilent Technologies). *C*q (“quantification cycle,” the cycle in which fluorescence from DNA amplification first exceeds background fluorescence) was determined at a fluorescence threshold of 0.23 for all runs; this fixed threshold was based on the average adaptive threshold of all individual runs. Amplification efficiencies for each primer pair were determined using the *C*q values obtained from a 1/4 dilution series (1:4, 1:16, 1:64, 1:256, and 1:1024) where *E* = 10^(1/−slope)^. Efficiency for each primer pair was calculated to be between 83 and 102% using the standard curve method (Table S6).

### Analysis of qPCR expression data

Before analysis, the *C*q values from qPCR were averaged over the three technical replicates, unless the replicates differed by >1 *C*q. In that case, the outlier technical replicate was removed and *C*q was averaged over the two remaining technical replicates. These averages were then both calibrator and efficiency normalized using the equation below. GAP amplified from the same sample of *M. l. luteus* young leaf cDNA acted as the interplate calibrator. Efficiency values for each gene are listed in Table S6. Relative expression of each gene was calculated as:

$$\mathrm{Relative}\;\mathrm{Expression}=\mathrm{Efficienc}y^{\Delta \mathrm{Cq}},\mathrm{where}\;\Delta \text{Cq}=\left(Cq^{\mathrm{calibrator}}\mathrm{-C}q^{\mathrm{sample}}\right)$$

In order to have a metric of gene stability that could be directly compared to stability estimates from RNA-seq data, the CV was calculated for each gene from the relative qPCR expression data. Calculations for mean expression (mean), SD, and for the CV (CV = SD/mean) were executed in Microsoft Excel. SD and mean were calculated from the relative expression of each of the four tissue types, averaged over the four biological replicates per tissue.

### Statistical analyses

All statistical tests were run using R software (Pumpkin Helmet, v.3.1.2). Linear models were fitted to obtain *t*-test results and Pearson’s correlation coefficient.

### Data availability

All transcriptomic expression data are provided in Table S7 and Table S8. Primer sequences are provided in Table S4. Raw reads from this study are deposited in Dryad (http://dx.doi.org/10.5061/dryad.84655) and are further analyzed in Edger *et al.* (2016).

## Results

### Identification of novel reference genes for Mimulus

In order to identify potential qPCR reference genes, we compared two simple methods for evaluating variation in expression across tissue types and growing environments: (a) genes with the lowest overall CV across all tissues from both transcriptome sets (T1 and T2; see Figure 1) and (b) exclusion of differentially expressed genes, determined through calculations of fold change, between pairwise comparisons of all tissue samples from both transcriptome sets. We identified 50 genes per species using the CV method and eight genes per species using the fold change method (Table S1 and Table S9) that have the potential to be good candidate reference genes for qPCR studies in *Mimulus*.

**Figure 2:**
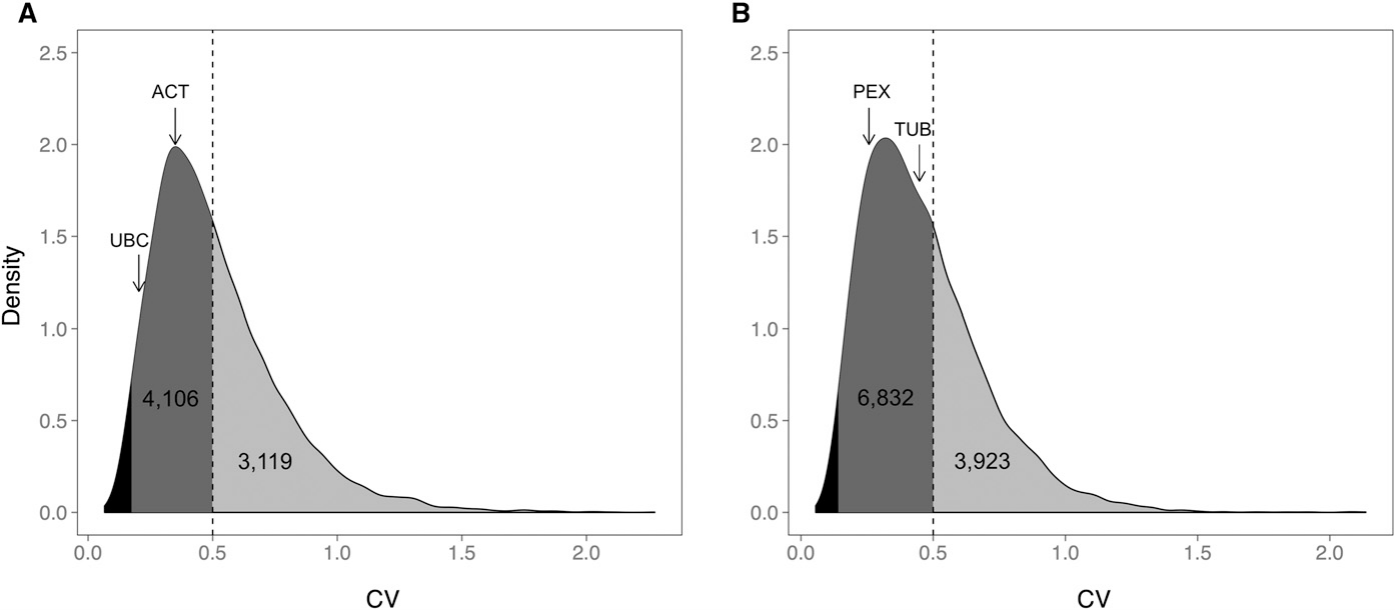
Distribution of CV for all reliably expressed genes (>5 FPKM in all samples) in (A) *M. guttatus* and (B) *M. l. luteus*. The dashed line marks the 0.50 CV cut-off for stably expressed genes and the arrows point to the two traditional reference genes with the lowest variation in expression for each species (Table S2). The portion of the density curves containing the top 200 genes with the lowest CV are shaded black; all genes selected using the CV and fold change method fall within this region. ACT, actin 2/7; CV, coefficient of variation; FPKM, fragments per kilobase per million reads; PEX, peroxin 4; TUB, β-tubulin 2; UBC, ubiquitin-conjugating enzyme.

Although CV was not correlated with total expression level (Figure S2), we used a minimum expression cut-off of 5 FPKM in order to exclude genes that are expressed at levels too low to be useful for qPCR normalization. The 50 genes with the lowest CV across both biological replicates of each species are listed in Table S9. Genes on this list have CVs <0.14 for *M. guttatus* and <0.12 for *M. l. luteus*. Although a 0.50 CV cut-off has previously been recommended for choosing qPCR reference genes (Hellemans *et al.* 2007), we find that the majority of robustly expressed genes fall under this cut-off (Figure 2). In *M. guttatus*, 4106 genes out of 7225 had a CV of <0.50; in *M. l. luteus*, 6832 genes out of 10,755 were under this cut-off.

For the fold change method, any genes with a log fold change >0.4 in *M. guttatus* or 0.3 in *M. l. luteus*, in any pairwise sample comparison, were excluded. Eight *M. guttatus* and eight *M. l. luteus* genes were identified in this manner that had low variation in expression across the four tissue types from two biological replicates (Table S1). The fold change method was consistent with the CV method; five *M. guttatus* genes and one *M. l. luteus* gene identified by the fold change method are also found on the top 50 CV list, and all of the genes identified by the fold change method are listed within the top 200 genes with the lowest CV (Table S10).

### Traditional reference genes in Mimulus

Since traditional reference genes can be inconsistently expressed in many biological systems (Brunner *et al.* 2004; Czechowski *et al.* 2005; Dheda *et al.* 2004; Suzuki *et al.* 2000), we investigated the expression variability of these traditional housekeeping reference genes in *Mimulus* using both transcriptomics and qPCR. We chose nine common traditional reference genes to analyze from the RNA-seq datasets: L8, ACT, ACT1, TUB, UBQ, UBC, PEX, GAP, and EF1 (see Table S2). We then corroborated the expression variability for four of these nine genes (ACT, PEX, UBC, and GAP) using qPCR (see Table S3).

**Figure 3:**
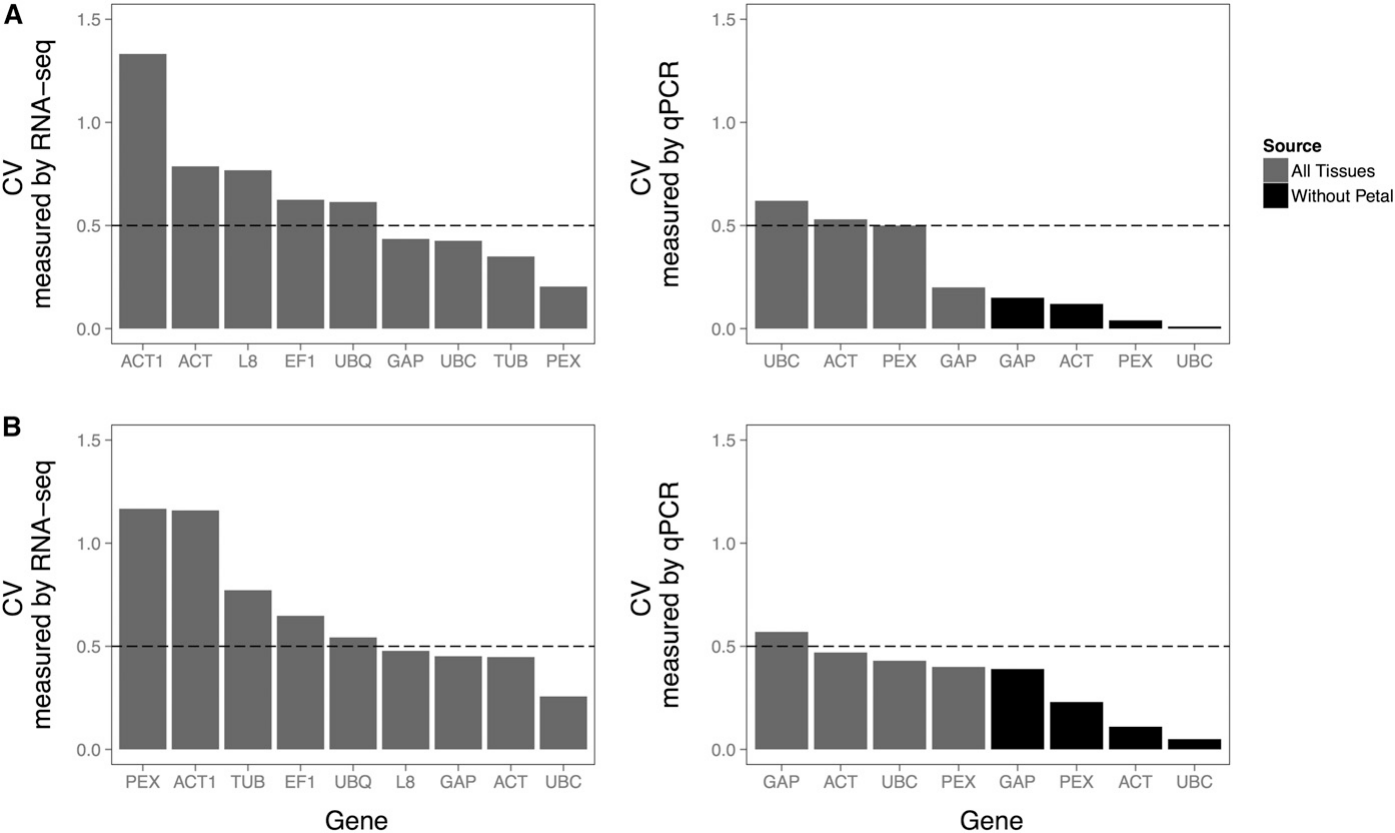
Expression variability estimates for selected traditional reference genes, based on coefficient of variation (CV). Expression variability in *M. guttatus* (A) and *M. l. luteus* (B) measured via RNA sequencing (RNA-seq) on both T1 and T2 (left column) or via quantitative polymerase chain reaction (qPCR) on T2 samples only (right column). Gray bars show the calculated CV when all tissue types are included and black bars show the calculated CV when petal tissue is excluded. Genes are ordered from most variable to least variable, with a dash line showing a previously suggested cut-off for usable reference genes at 0.50 CV. For the tetraploid *M. l. luteus*, CVs reported for the RNA-seq data are the average of both homeologs. The genes tested include 60s ribosomal protein L8 (L8), actin 2/7 (ACT), actin 11 (ACT1), β-tubulin 2 (TUB), ubiquitin 5 (UBQ), ubiquitin conjugating enzyme 25 (UBC), peroxin 4 (PEX), GAPDH-C1 (GAP), and EF1-α (EF1). CV was calculated from fragments per kilobase per million reads values for genes measured via RNA-seq and from relative expression values, calculated by Efficiency^Δ^*^C^*^q^, where Δ*C*q = *C*q_Calibrator_ – *C*q_sample_, for genes measured via qPCR.

In both *M. guttatus* and *M. l. luteus*, there were thousands of expressed genes with lower CVs than the traditional housekeeping genes (Figure 2 and Table S2), and none of the traditional housekeeping genes were among the 16 genes identified by the fold change method. Nevertheless, four traditional genes in *M. guttatus* (GAP, UBC, TUB, and PEX) and four in *M. l. luteus* (L8, GAP, ACT, and UBC) do have CVs <0.5, suggesting that they could be useful reference genes for qPCR normalization in these species (Figure 3).

The follow-up qPCR validation reported much lower expression variability for the tested subset of traditional genes. This is most likely due to a less variable group of plants being measured for qPCR than were measured for RNA-seq (see Figure 1). Expression variability was even lower when measures from petal tissue were excluded (Figure 3), as expression levels for all four tested genes were substantially higher in petal tissue than in the other three tissue types (Figure S3). This is only the case for the qPCR data and there is no trend in the RNA-seq data when petal is excluded, even though transcriptome T2 was derived from one of the same RNA samples that was used for qPCR. When all tissues were included in the qPCR variability calculations, we found that GAP had the lowest variation in expression in *M. guttatus* and PEX was the least variable in *M. l. luteus*. When petal was excluded, UBC was the least variable traditional reference gene in both species.

### Efficacy of transcriptomics for reference gene selection

Although environmental condition was not a purposeful manipulation in our study, the different growth histories of the genetically identical plants used for the two *M. l. luteus* transcriptome sets allowed us to evaluate the robustness of gene expression to moderate environmental variation. This was achieved by comparing both mean expression and expression variability (measured by CV) across the different tissue types between T1 and T2. For comparison, we also evaluated the two *M. guttatus* transcriptome sets, although the plants used in this comparison were genetically as well as environmentally different (see Figure 1).

**Figure 4:**
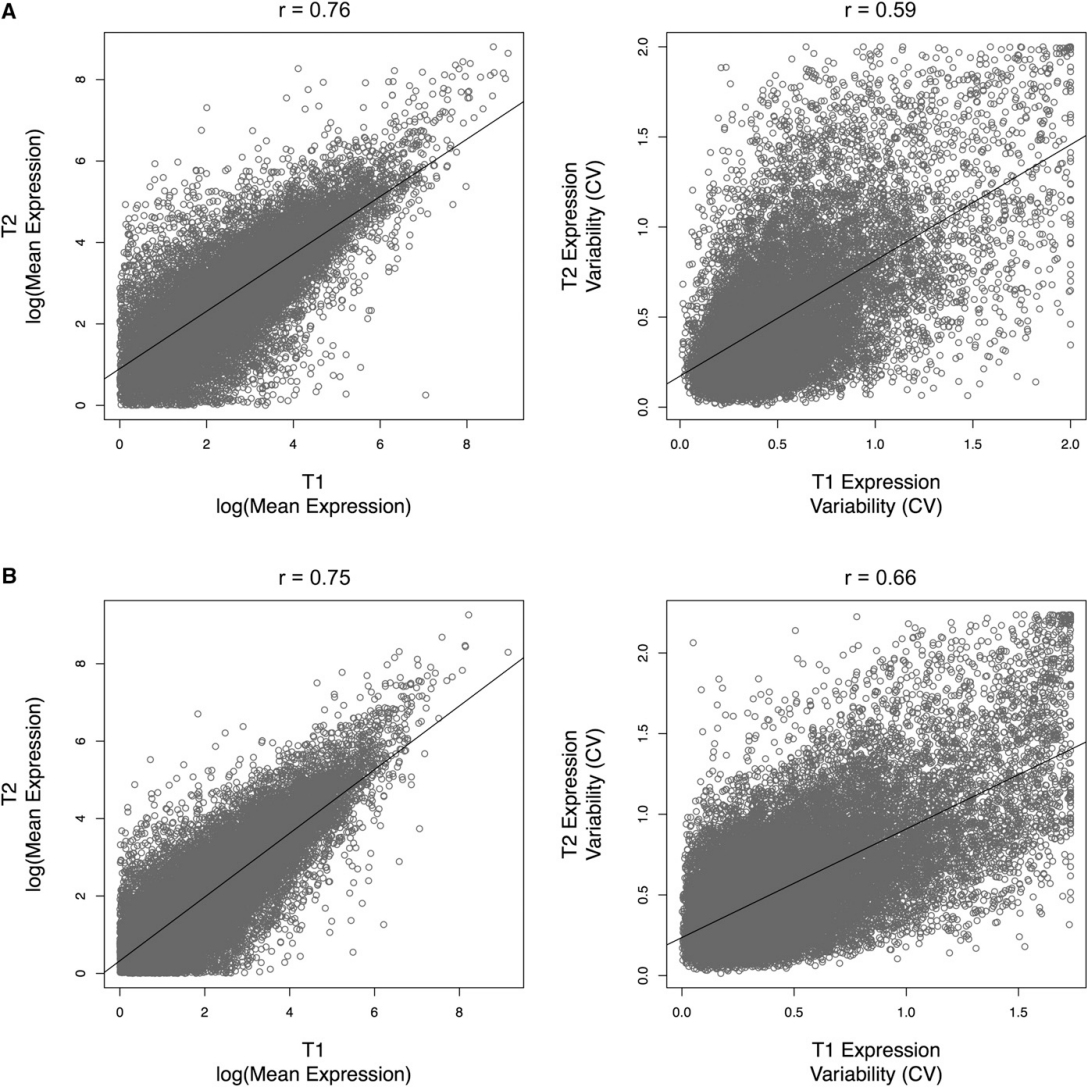
Correlation in expression mean (FPKM) and variability (CV), as measured by RNA-seq, between the biological replicates (T1 and T2) of *M. guttatus* (A) and *M. l. luteus* (B). Values for Pearson’s correlation coefficient are given above each graph and the line of best fit is shown in black. Genes with mean expression <1 FPKM were excluded from the plot. CV, coefficient of variation; FPKM, fragments per kilobase per million reads; RNA-seq, RNA sequencing.

The correlation in CV between T1 and T2 is weaker than the correlation in mean expression for both species, showing a stronger environmental effect on the variance than on the mean (Figure 4). Additionally, CV estimates were more closely correlated between the replicates of *M. l. luteus* than between the replicates of *M. guttatus*, as expected given that the *M. l. luteus* replicates came from the same highly inbred line of plants while the *M. guttatus* replicates came from different lineages.

**Figure 5:**
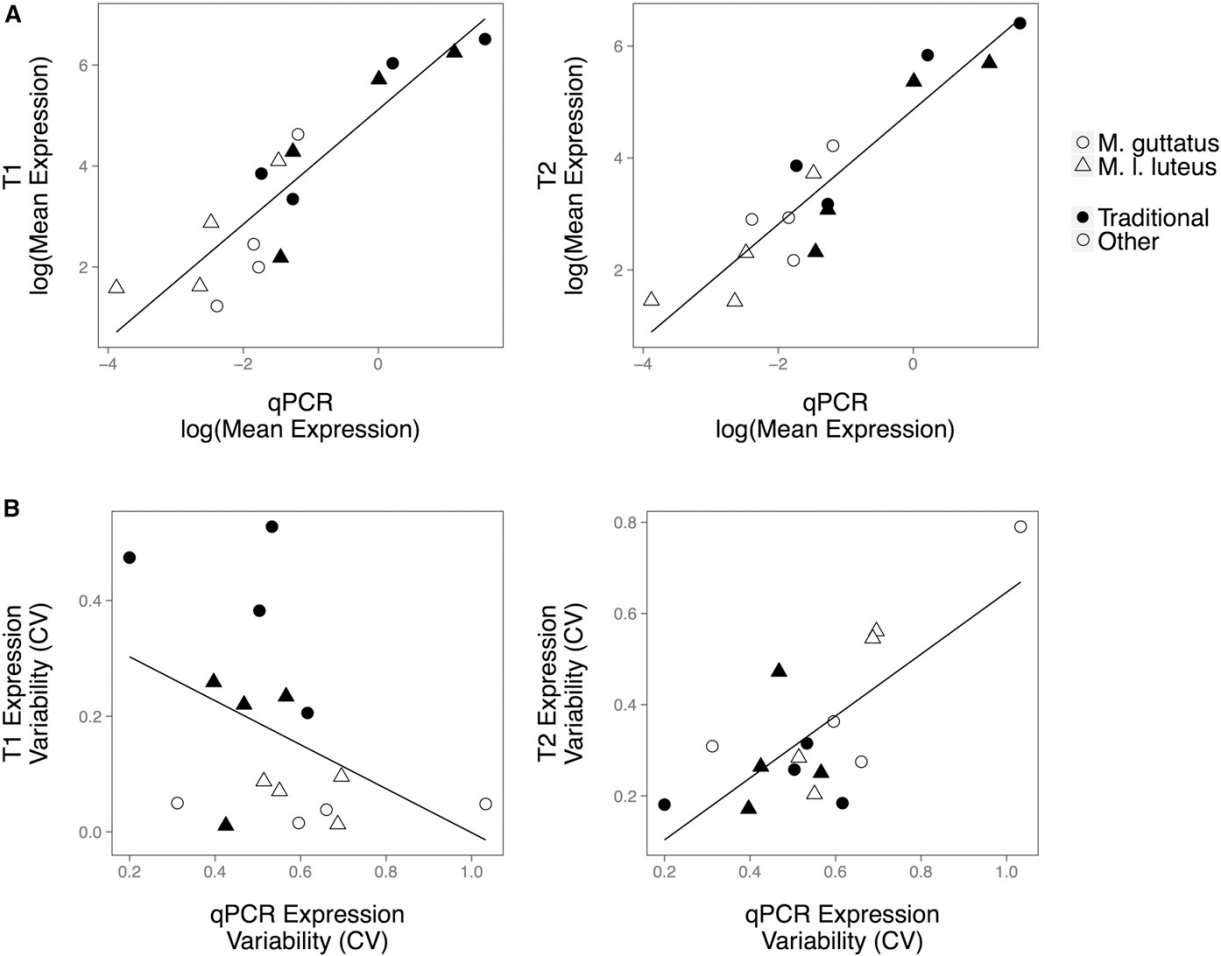
Comparisons of relative gene expression and of expression variability as determined by RNA-seq and qPCR for a sample of four traditional reference genes (closed symbols) and four additional genes that had initially been found to be stably expressed in transcriptome T1 (open symbols) (Table S3). The T2 RNA-seq transcriptomes and the qPCR data were derived from genetically identical plants grown in the same greenhouse, while the T1 RNA-seq transcriptomes were derived from plants grown in a greenhouse at a separate institution (Figure 1). (A) There is a strong correlation in relative expression determined by qPCR and RNA-seq, for both T1 (left panel, *r* = 0.90, *P* < 0.001) and T2 (right panel, *r* = 0.85, *P* < 0.001). (B) Expression variability (CV) measured via qPCR is correlated with expression variability measured via T2 (right panel, *r* = 0.74, *P* = 0.001), but is not correlated to expression variability measured via T1 (left panel, *r* = −0.42, *P* = 0.104). Expression data from both *M. guttatus* and *M. l. luteus* are included together. Relative expression of T1 and T2 is given in FPKM. Relative qPCR expression = Efficiency^Δ^*^C^*^q^, where Δ*C*q = *C*q_Calibrator_ − *C*q_sample_. CV, coefficient of variation; FPKM, fragments per kilobase per million reads; qPCR, quantitative polymerase chain reaction; RNA-seq, RNA sequencing.

The expression data collected via RNA-seq were validated for accuracy using qPCR expression data for a selected group of eight genes, including four traditional reference genes (Table S3). Mean expression values measured by RNA-seq transcriptomes T1 and T2 were both in agreement with values found via qPCR (Figure 5A). In contrast, expression variability estimated by qPCR was significantly correlated with T2 expression variability, but had no significant relationship to T1 expression variability (Figure 5B). This is most likely due to variation in plant lineage and plant growth conditions, as the T2 transcriptomes and the qPCR data derive from genetically identical plants that were grown in the same greenhouse, while the T1 transcriptomes derive from plants grown in a greenhouse at a separate institution. For *M. guttatus*, T1 and T2 also differed in the accession used (Figure 1). This pattern, particularly for the isogenic *M. l. luteus* transcriptomes, suggests that environmental factors may have a greater effect on the “noise” in gene expression than on the expression level itself.

## Discussion

### Identification of novel reference genes for Mimulus

While RNA-seq has the potential to accurately identify genes with low variation in expression, there is still not a universally accepted method for selecting reference genes from RNA-seq data. Most of the programs that are widely used for reference gene selection, such as geNorm, BestKeeper, and NormFinder, were designed specifically for qPCR data and can only process a handful of genes at a time (Vandesompele *et al.* 2002; Andersen *et al.* 2004; Pfaffl *et al.* 2004). We explored two different methods for identifying stably expressed genes from whole-transcriptome data: (1) ranking genes based on the CV of expression across different samples (CV method) and (2) excluding unstable genes using a log fold change cut-off value (fold change method). We find that both methods identify many stably expressed genes that have the potential to be novel reference genes for qPCR expression studies in *M. guttatus* and *M. l. luteus* (see Table S1 and Table S9).

Using the CV method, all expressed genes from *M. guttatus* and *M. l. luteus* were ranked based on the variability of their expression across different tissue types and growing conditions, and the top 50 genes with the lowest variability were identified (Table S9). Using the fold change method, we identified eight *M. guttatus* and eight *M. l. luteus* genes with low variability in expression across four different tissue types and two biological replicates. No traditional reference genes were identified as being among the top 50 most stably expressed genes by either of our methods. In addition, the novel reference genes we identified had much lower expression variability in our system than any of the most commonly used traditional reference genes (Figure 2), which highlights the utility of the whole-transcriptome approach to reference gene selection.

The advantage of using either of these methods for reference gene selection is their simplicity in calculation. While the fold change method has the benefit of producing a discrete list of genes with low variation in expression, the CV method has the benefit of quantifying expression variability in a way where genes can be ranked and directly compared. These methods have previously been used in other plant species to select novel reference genes from transcriptomic data (Czechowski *et al.* 2005; Chang *et al.* 2012), but we are the first to show that these two methods produce comparable results. All of the genes found on the fold change short-list were among the 200 genes with the lowest CV, which corresponds to the top 2–3% most stably expressed transcripts. Ideally, novel reference genes would be selected that score well according to both metrics.

The CVs of the novel reference genes we identified are all <0.20, whereas a previously suggested cut-off for valid reference genes is a CV of 0.50 (Hellemans *et al.* 2007). It is important to note that using a 0.50 CV cut-off in our system included the majority of expressed genes (Figure 2), and thus it was not a very discriminatory standard for determining expression variability.

### Traditional reference genes in Mimulus

Many studies have pointed to the instability of traditional housekeeping reference genes (Brunner *et al.* 2004; Czechowski *et al.* 2005; Dheda *et al.* 2004; Suzuki *et al.* 2000). We find that some traditional reference genes in *Mimulus* have the potential to work well for qPCR normalization. Using a whole-transcriptome method, we identified four traditional reference genes that have somewhat low variation in expression (CV < 0.50) in *M. guttatus* and *M. l. luteus* (Figure 3). Two genes, UBC and GAP, were even identified as stably expressed in both species and could potentially be good universal reference genes for the *Mimulus* genus. We confirmed our findings for four of these traditional reference genes with qPCR and found that all four (GAP, ACT, UBC, and PEX) could be acceptable as reference genes for both species based on qPCR estimates of expression variability across tissues, although some of the genes were at or slightly above the recommended 0.5 CV cut-off when the relatively divergent petal tissue samples were included (Figure 3). However, these traditional reference genes were nowhere near the most stably expressed in the transcriptome as a whole (Figure 2), which highlights the opportunity to discover dramatically more stable reference genes using a transcriptome-guided approach.

Despite the widespread use of *Mimulus* as a model genus for genetics, very few papers have attempted to validate reference genes for use in this genus. As part of a larger study, Scoville *et al.* (2011) qualitatively ranked the expression variability of six traditional reference genes in *M. guttatus* and found that UBQ and EF1 were the most stably expressed. We quantitatively investigated four of these six traditional reference genes in our own study and found that UBQ and EF1 had higher expression variability than other traditional reference genes and that, in both species, the genes' CVs were >0.5 under our study conditions. Scoville *et al.* (2011) tested different lines of *M. guttatus* and included a wound treatment, which may have resulted in our differing reports of traditional reference gene stabilities. This again highlights the importance of reference gene validation for specific study conditions.

Although we found that some traditional reference genes can be used for qPCR normalization, they are not optimal reference genes; the variability in expression of the traditional reference genes is very high when compared to the variability of all robustly expressed genes (Figure 2 and Table S2). This indicates that whole-transcriptome approaches, such as RNA-seq, have great potential to discover novel reference genes that are stably expressed in the study system of interest. With the current speed and low cost of RNA-seq, as well as the online availability of multi-tissue and/or multi-environment RNA-seq data sets, we expect that the whole-transcriptome approach will be increasingly useful for reference gene identification and validation.

### Efficacy of transcriptomics for reference gene selection

RNA-seq has been repeatedly shown to generate accurate measurements of gene expression (Marioni *et al.* 2008; Mortazavi *et al.* 2008; Nagalakshmi *et al.* 2008; Nookaew *et al.* 2012). We find similar results in *Mimulus* when comparing the relative expression determined by RNA-seq to the relative expression determined by qPCR for eight selected genes (Figure 5A). We also find that estimates of expression mean are robust to moderate environmental and genetic variation, but that estimates of expression variability across tissue types are only in agreement when the samples are obtained from a shared environment (Figure 4 and Figure 5). These results suggest that environmental changes may have a greater impact on expression variability than on expression means.

For the goal of reference gene selection, where expression variability must remain low, this difficulty can be solved in two ways. One approach is to use the same samples for both RNA-seq and the subsequent qPCR analysis, as in Chang *et al.* (2012) and Yang *et al.* (2014). This method would be highly accurate, but would be extremely specific to particular study conditions. A second approach would be to evaluate a large variety of genotypes or growth conditions to discover genes that are maximally stable across genetically and environmentally distinct samples, as was done for *A. thaliana* in Czechowski *et al.* (2005). This method would allow for the identification of a starting pool of “universally” stable genes.

### Reference gene selection using RNA-seq

We show, using *Mimulus* as a case study, that RNA-seq is a promising tool for selecting genes with low gene expression variance that can be used as novel qPCR reference genes. As many research labs regularly use RNA-seq as a first approach to collecting expression data, already completed RNA-seq transcriptomes are a readily available tool that can be used to search for candidate qPCR reference genes in any study system. Although we find that the variance in expression is variable between environmental conditions, we propose that transcriptomes from diverse samples can be pooled in order to identify more universally stable genes. We show that two simple methods for identifying genes with low expression variance, the CV method and the fold change method, both result in comparable evaluations of expression variance. Thus, either of these methods can be used to identify a preliminary set of highly stable candidate reference genes for qPCR experiments.

## Supplementary Material

Supplemental material is available online at www.g3journal.org/lookup/suppl/doi:10.1534/g3.116.038075/-/DC1.

## References

[bib1] Andersen C. L. Jensen J. L. Orntoft T. F., 2004 Normalization of real-time quantitative reverse transcription-PCR data: a model-based variance estimation approach to identify genes suited for normalization, applied to bladder and colon cancer data sets. Cancer Res. 64(15): 5245–5250. 15289330 10.1158/0008-5472.CAN-04-0496

[bib2] Barker W. Nesom G. Beardsley P. M. Fraga N. S., 2012 A taxonomic conspectus of Phrymaceae: a narrowed circumscription for *Mimulus*, new and resurrected genera, and new names and combinations. Phytoneuron 39: 1–60.

[bib3] Beardsley P. M. Olmstead R. G., 2002 Redefining Phrymaceae: the placement of *Mimulus*, tribe Mimuleae and Phryma. Am. J. Bot. 89(7): 1093–1102. 21665709 10.3732/ajb.89.7.1093

[bib4] Brunner A. M. Yakovlev I. A. Strauss S. H., 2004 Validating internal controls for quantitative plant gene expression studies. BMC Plant Biol. 4: 14. 15317655 10.1186/1471-2229-4-14 PMC515301

[bib5] Bustin S. A. Benes V. Garson J. A. Hellemans J. Huggett J., 2009 The MIQE guidelines: minimum information for publication of quantitative real-time PCR experiments. Clin. Chem. 55(4): 611–622. 19246619 10.1373/clinchem.2008.112797

[bib6] Chang E. M. Shi S. Q. Liu J. F. Cheng T. L. Xue L., 2012 Selection of reference genes for quantitative gene expression studies in *Platycladus orientalis* (Cupressaceae) using Real-Time PCR. PLoS One 7(3): e33278. 22479379 10.1371/journal.pone.0033278 PMC3316566

[bib7] Cooley A. M. Carvallo G. Willis J. H., 2008 Is floral diversification associated with pollinator divergence? Flower shape, flower colour and pollinator preference in Chilean *Mimulus*. Ann. Bot. (Lond.) 101(5): 641–650. 10.1093/aob/mcn014 PMC2710177 18272528

[bib8] Czechowski T. Stitt M. Altmann T. Udvardi M. K. Scheible W. R., 2005 Genome-wide identification and testing of superior reference genes for transcript normalization in *Arabidopsis*. Plant Physiol. 139(1): 5–17. 16166256 10.1104/pp.105.063743 PMC1203353

[bib9] de Oliveira L. A. Breton M. C. Bastolla F. M. Camargo S. D. Margis R., 2012 Reference genes for the normalization of gene expression in *Eucalyptus* species. Plant Cell Physiol. 53(2): 405–422. 22197885 10.1093/pcp/pcr187 PMC7107212

[bib10] Dheda K. Huggett J. F. Bustin S. A. Johnson M. A. Rook G., 2004 Validation of housekeeping genes for normalizing RNA expression in real-time PCR. Biotechniques 37(1): 112–119. 15283208 10.2144/04371RR03

[bib11] Dheda K. Huggett J. F. Chang J. S. Kim L. U. Bustin S. A., 2005 The implications of using an inappropriate reference gene for real-time reverse transcription PCR data normalization. Anal. Biochem. 344(1): 141–143. 16054107 10.1016/j.ab.2005.05.022

[bib12] Edger, P. P., R. Smith, M. R. McKain, A. M. Cooley, M. Vallejo-Marin *et al.*, 2016 Subgenome dominance in an interspecific hybrid, synthetic allopolyploid, and a 140 year old naturally established neo-allopolyploid monkeyflower. bioRxiv. Available at: https://doi.org/10.1101/094797. 10.1105/tpc.17.00010 PMC5635986 28814644

[bib13] Grabherr M. G. Haas B. J. Yassour M. Levin J. Z. Thompson D. A., 2011 Full-length transcriptome assembly from RNA-Seq data without a reference genome. Nat. Biotechnol. 29(7): 644–652. 21572440 10.1038/nbt.1883 PMC3571712

[bib14] Gutierrez L. Mauriat M. Guenin S. Pelloux J. Lefebvre J. F., 2008 The lack of a systematic validation of reference genes: a serious pitfall undervalued in reverse transcription-polymerase chain reaction (RT-PCR) analysis in plants. Plant Biotechnol. J. 6(6): 609–618. 18433420 10.1111/j.1467-7652.2008.00346.x

[bib15] Haas B. J. Zody M. C., 2010 Advancing RNA-Seq analysis. Nat. Biotechnol. 28(5): 421–423. 20458303 10.1038/nbt0510-421

[bib16] Hellemans J. Mortier G. De Paepe A. Speleman F. Vandesompele J., 2007 qBase relative quantification framework and software for management and automated analysis of real-time quantitative PCR data. Genome Biol. 8(2): R19. 17291332 10.1186/gb-2007-8-2-r19 PMC1852402

[bib17] Hellsten U. Wright K. M. Jenkins J. Shu S. Q. Yuan Y. W., 2013 Fine-scale variation in meiotic recombination in *Mimulus* inferred from population shotgun sequencing. Proc. Natl. Acad. Sci. USA 110(48): 19478–19482. 24225854 10.1073/pnas.1319032110 PMC3845195

[bib18] Libault M. Thibivilliers S. Bilgin D. D. Radwan O. Benitez M., 2008 Identification of four soybean reference genes for gene expression normalization. Plant Genome 1(1): 44–54.

[bib19] Marioni J. C. Mason C. E. Mane S. M. Stephens M. Gilad Y., 2008 RNA-seq: an assessment of technical reproducibility and comparison with gene expression arrays. Genome Res. 18(9): 1509–1517. 18550803 10.1101/gr.079558.108 PMC2527709

[bib20] Mortazavi A. Williams B. A. McCue K. Schaeffer L. Wold B., 2008 Mapping and quantifying mammalian transcriptomes by RNA-Seq. Nat. Methods 5(7): 621–628. 18516045 10.1038/nmeth.1226 PMC13303166

[bib21] Mukherjee B. B. Vickery R. K., 1962 Chromosome counts in the section *Simiolus* of the genus *Mimulus* (Scrophulariaceae). V. The chromosomal homologies of *M. guttatus* and its allied species and varieties. Madrono 16: 141–172.

[bib22] Nagalakshmi U. Wang Z. Waern K. Shou C. Raha D., 2008 The transcriptional landscape of the yeast genome defined by RNA sequencing. Science 320(5881): 1344–1349. 18451266 10.1126/science.1158441 PMC2951732

[bib23] Nookaew I. Papini M. Pornputtapong N. Scalcinati G. Fagerberg L., 2012 A comprehensive comparison of RNA-Seq-based transcriptome analysis from reads to differential gene expression and cross-comparison with microarrays: a case study in *Saccharomyces cerevisiae*. Nucleic Acids Res. 40(20): 10084–10097. 22965124 10.1093/nar/gks804 PMC3488244

[bib24] Pfaffl M. W. Tichopad A. Prgomet C. Neuvians T. P., 2004 Determination of stable housekeeping genes, differentially regulated target genes and sample integrity: BestKeeper – Excel-based tool using pair-wise correlations. Biotechnol. Lett. 26(6): 509–515. 15127793 10.1023/b:bile.0000019559.84305.47

[bib25] Robertson G. Schein J. Chiu R. Corbett R. Field M., 2010 *De novo* assembly and analysis of RNA-seq data. Nat. Methods 7(11): 909–911. 20935650 10.1038/nmeth.1517

[bib26] Robinson M. D. McCarthy D. J. Smyth G. K., 2010 edgeR: a Bioconductor package for differential expression analysis of digital gene expression data. Bioinformatics 26(1): 139–140. 19910308 10.1093/bioinformatics/btp616 PMC2796818

[bib27] Scoville A. G. Barnett L. L. Bodbyl-Roels S. Kelly J. K. Hileman L. C., 2011 Differential regulation of a MYB transcription factor is correlated with transgenerational epigenetic inheritance of trichome density in *Mimulus guttatus*. New Phytol. 191(1): 251–263. 21352232 10.1111/j.1469-8137.2011.03656.x PMC3107365

[bib28] Simão F. A. Waterhouse R. M. Ioannidis P. Kriventseva E. V. Zdobnov E. M., 2015 BUSCO: assessing genome assembly and annotation completeness with single-copy orthologs. Bioinformatics 19: 3210–3212. 10.1093/bioinformatics/btv351 26059717

[bib29] Sobel J. M. Streisfeld M. A., 2013 Flower color as a model system for studies of plant evo-devo. Front. Plant Sci. 4(321): 1–17. 23970892 10.3389/fpls.2013.00321 PMC3748380

[bib30] Suzuki T. Higgins P. J. Crawford D. R., 2000 Control selection for RNA quantitation. Biotechniques 29(2): 332–337. 10948434 10.2144/00292rv02

[bib31] Thellin O. Zorzi W. Lakaye B. De Borman B. Coumans B., 1999 Housekeeping genes as internal standards: use and limits. J. Biotechnol. 75(2–3): 291–295. 10617337 10.1016/s0168-1656(99)00163-7

[bib32] Trapnell C. Williams B. A. Pertea G. Mortazavi A. Kwan G., 2010 Transcript assembly and quantification by RNA-Seq reveals unannotated transcripts and isoform switching during cell differentiation. Nat. Biotechnol. 28(5): 511–515. 20436464 10.1038/nbt.1621 PMC3146043

[bib33] Twyford A. D. Streisfeld M. A. Lowry D. B. Friedman J., 2015 Genomic studies on the nature of species: adaptation and speciation in *Mimulus*. Mol. Ecol. 24(11): 2601–2609. 25856725 10.1111/mec.13190

[bib34] Vallejo‐Marín M. Buggs R. J. Cooley A. M. Puzey J. R., 2015 Speciation by genome duplication: repeated origins and genomic composition of the recently formed allopolyploid species *Mimulus peregrinus*. Evolution 69: 1487–1500. 25929999 10.1111/evo.12678 PMC5033005

[bib35] Vandesompele J. De Preter K. Pattyn F. Poppe B. Van Roy N., 2002 Accurate normalization of real-time quantitative RT-PCR data by geometric averaging of multiple internal control genes. Genome Biol. 3(7): RESEARCH0034. 12184808 10.1186/gb-2002-3-7-research0034 PMC126239

[bib36] Wang Z. Gerstein M. Snyder M., 2009 RNA-Seq: a revolutionary tool for transcriptomics. Nat. Rev. Genet. 10(1): 57–63. 19015660 10.1038/nrg2484 PMC2949280

[bib37] Willis J. H., 1999 The role of genes of large effect on inbreeding depression in *Mimulus guttatus*. Evolution 53(6): 1678–1691. 10.1111/j.1558-5646.1999.tb04553.x 28565461

[bib38] Wu C. A. Lowry D. B. Cooley A. M. Wright K. M. Lee Y. W., 2008 *Mimulus* is an emerging model system for the integration of ecological and genomic studies. Heredity (Edinb) 100(2): 220–230. 17551519 10.1038/sj.hdy.6801018

[bib39] Yang H. L. Liu J. Huang S. M. Guo T. T. Deng L. B., 2014 Selection and evaluation of novel reference genes for quantitative reverse transcription PCR (qRT-PCR) based on genome and transcriptome data in *Brassica napus* L. Gene 538(1): 113–122. 24406618 10.1016/j.gene.2013.12.057

[bib40] Yuan Y. W. Sagawa J. M. Young R. C. Christensen B. J. Bradshaw H. D., 2013 Genetic dissection of a major anthocyanin QTL contributing to pollinator-mediated reproductive isolation between sister species of *Mimulus*. Genetics 194: 255–263. 23335333 10.1534/genetics.112.146852 PMC3632473

[bib41] Zhuang H. Fu Y. He W. Wang L. Wei Y., 2015 Selection of appropriate reference genes for quantitative real-time PCR in *Oxytropis ochrocephala* Bunge using transcriptome datasets under abiotic stress treatments. Front. Plant Sci. 6: 475. 26175743 10.3389/fpls.2015.00475 PMC4484982

