# Supplementary material for: A Whole-Transcriptome Approach to Evaluating Reference Genes for Quantitative Gene Expression Studies: A Case Study in Mimulus
Source: G3 (Bethesda). 2017 Mar 3;7(4):1085–95. doi: 10.1534/g3.116.038075 (PMC5386857; doi:10.1534/g3.116.038075)
Supplement: Supplementary file 1 [file 1085FigureS1.docx]

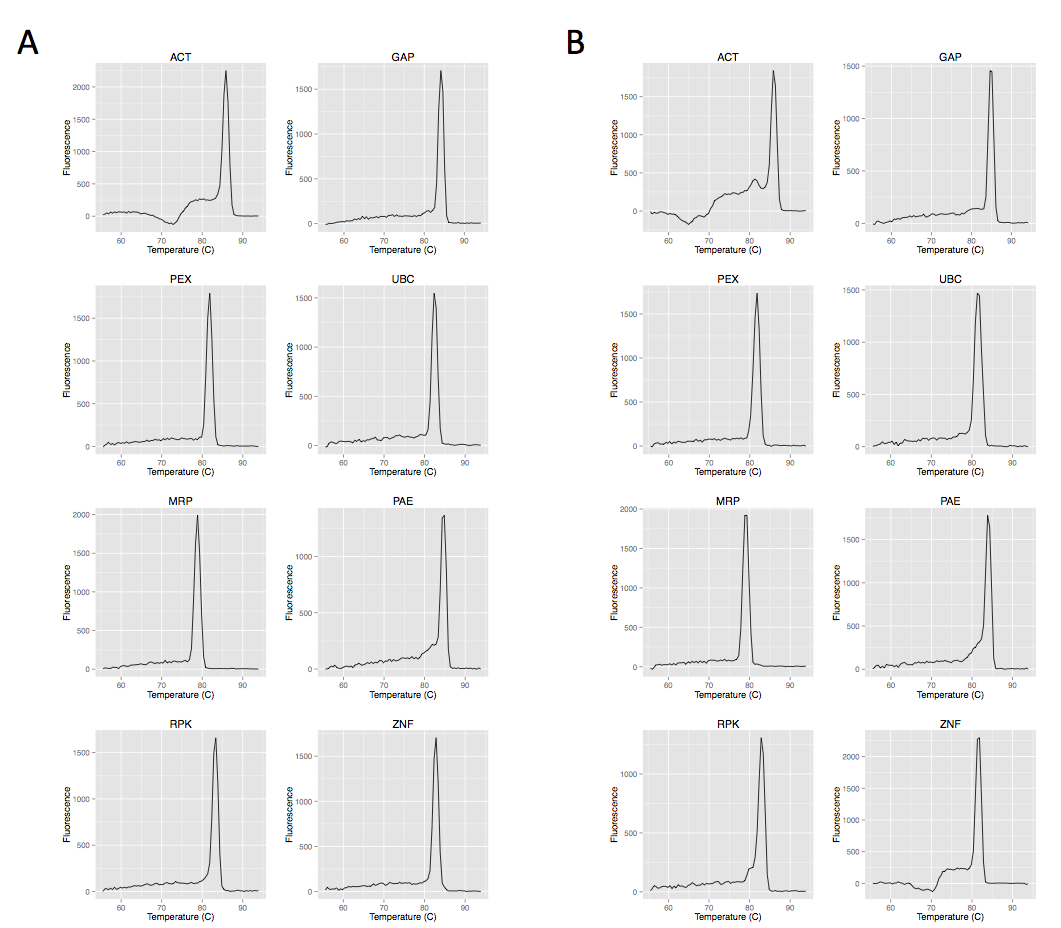


**Figure S1.** Dissociation curves for the select eight genes tested via qPCR in (A) *M. guttatus* and (B) *M. l. luteus.* See Table S4 for the full list of primer pairs used for amplification and for the definition of gene acronyms. Dissociation curves were taken from one random sample of the 16 total samples measured via qPCR.
