# Supplementary material for: A Whole-Transcriptome Approach to Evaluating Reference Genes for Quantitative Gene Expression Studies: A Case Study in Mimulus
Source: G3 (Bethesda). 2017 Mar 3;7(4):1085–95. doi: 10.1534/g3.116.038075 (PMC5386857; doi:10.1534/g3.116.038075)
Supplement: Supplementary file 2 [file 1085FigureS2.docx]

**Figure S2**. The coefficient of variation (CV) of gene expression across different tissue types is not higher for genes with low mean expression levels. Although there is a significant correlation between mean expression and CV for both species (p < 0.001), the effect of the association is minor in both *M. guttatus* (m = -0.015) and *M. l. luteus* (m = -0.018). Mean expression was calculated over both biological replicates per species.
