# Supplementary material for: A Whole-Transcriptome Approach to Evaluating Reference Genes for Quantitative Gene Expression Studies: A Case Study in Mimulus
Source: G3 (Bethesda). 2017 Mar 3;7(4):1085–95. doi: 10.1534/g3.116.038075 (PMC5386857; doi:10.1534/g3.116.038075)
Supplement: Supplementary file 3 [file 1085FigureS3.docx]

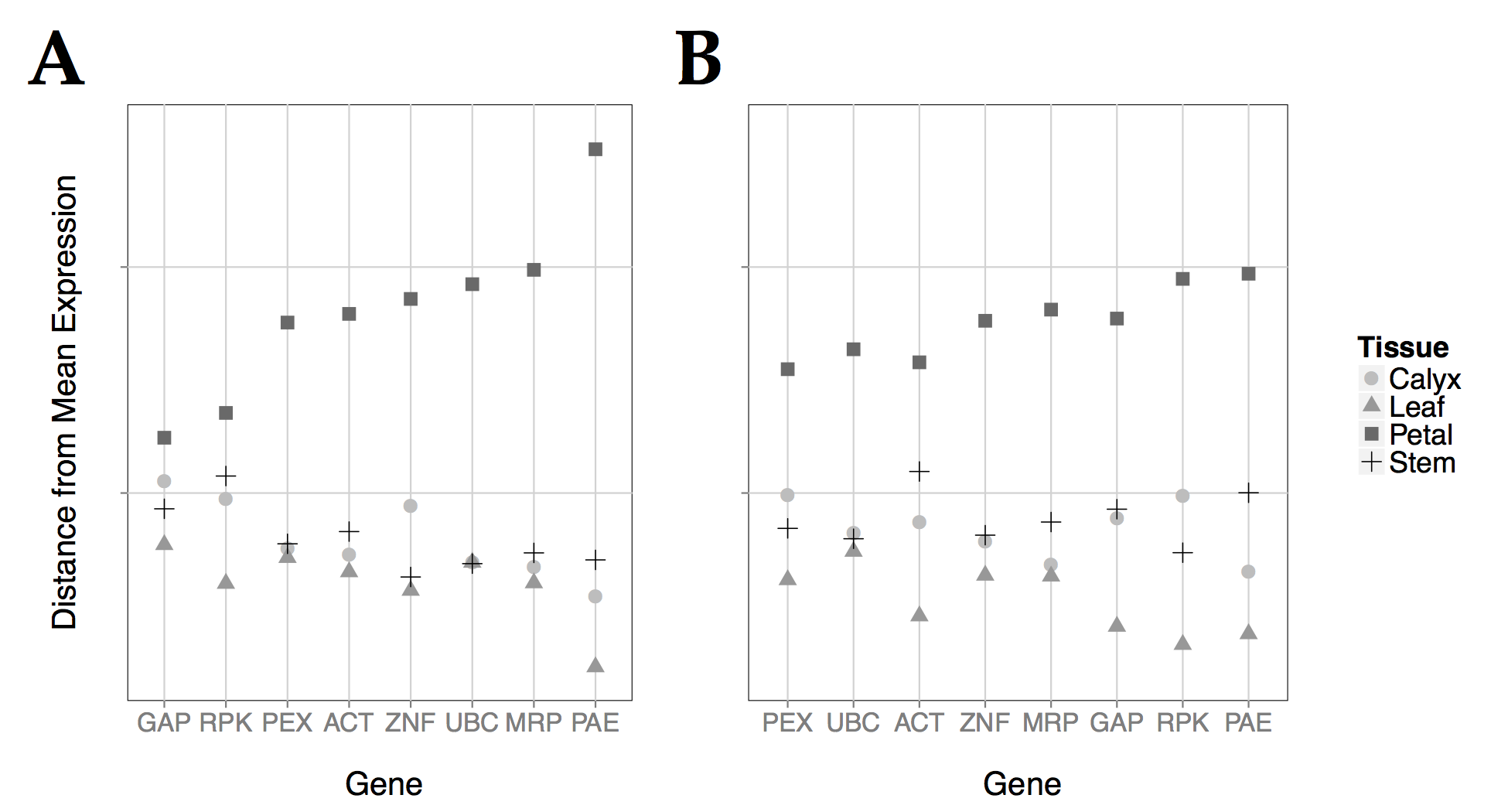


**Figure S3**. qPCR gene expression by tissue type. For every gene tested, petal tissue shows the highest mean expression level for both *M. guttatus* (A) and *M. l. luteus* (B). Genes from each species are ordered from most stable (left) to least stable (right) based on CV values. Each point represents average expression in that tissue across the four biological replicates. Distance from mean expression was calculated using the following equation: (tissue expression – mean expression)/mean expression.
