# Supplementary material for: A Whole-Transcriptome Approach to Evaluating Reference Genes for Quantitative Gene Expression Studies: A Case Study in Mimulus
Source: G3 (Bethesda). 2017 Mar 3;7(4):1085–95. doi: 10.1534/g3.116.038075 (PMC5386857; doi:10.1534/g3.116.038075)
Supplement: Supplementary file 5 [file 1085TableS2.docx]

**Table S2. Expression variability of nine traditional reference genes in *M. guttatus* and *M. l. luteus*.**

| Species | Gene | Annotation | Arabidopsis ortholog | Mean^a^ | SD | CV^b^ | Rank^c^ |
| --- | --- | --- | --- | --- | --- | --- | --- |
|  |  |  |  |  |  |  |  |
| *M. guttatus* | PEX | Peroxin 4 | NM_122477.2 | 26.2 | 5.3 | 0.20 | 418 |
|  | TUB | β-Tubulin 2 | NM_125664.3 | 640.6 | 224.2 | 0.35 | 2,684 |
|  | UBC | Ub conjugating 25 | NM_112402.2 | 34.2 | 14.5 | 0.42 | 4,299 |
|  | GAP | GAPDH C1 | NM_106601.3 | 380.6 | 165.7 | 0.43 | 4,525 |
|  | UBQ | Ubiquitin 5 | J05539.1 | 83.2 | 51.1 | 0.61 | 8,321 |
|  | EF1 | EF1α-1 | NM_001035916.2 | 35.7 | 22.3 | 0.62 | 8,530 |
|  | L8 | 60S ribosomal L8 | NM_119780.2 | 522.3 | 400.9 | 0.76 | 11,009 |
|  | ACT | Actin 2/7 | AY120779.1 | 200.4 | 157.7 | 0.78 | 11,330 |
|  | ACT1 | Actin 11 | U27981.1 | 139.4 | 185.7 | 1.33 | 17,593 |
|  |  |  |  |  |  |  |  |
| *M. l. luteus* | UBC | Ub conjugating 25 | NM_112402.2 | 9.8 | 2.5 | 0.25 | 3,298 |
|  | ACT | Actin 2/7 | AY120779.1 | 290.1 | 130.0 | 0.44 | 10,255 |
|  | GAP | GAPDH C1 | NM_106601.3 | 297.4 | 134.6 | 0.45 | 5,591 |
|  | L8 | 60S ribosomal L8 | NM_119780.2 | 154.4 | 73.8 | 0.47 | 11,090 |
|  | UBQ | Ubiquitin 5 | J05539.1 | 132.8 | 72.0 | 0.54 | 14,264 |
|  | EF1 | EF1α-1 | NM_001035916.2 | 16.7 | 10.8 | 0.64 | 3,423 |
|  | TUB | β-Tubulin 2 | NM_125664.3 | 65.3 | 50.4 | 0.77 | 24,272 |
|  | ACT1 | Actin 11 | U27981.1 | 74.5 | 86.4 | 1.15 | 32,733 |
|  | PEX | Peroxin 4 | NM_122477.2 | 11.2 | 13.1 | 1.16 | 21,924 |

^a^ Mean expression measured in FPKM. ^b^ CV calculated using the equation CV = SD/Mean. ^c^ Rank is the relative ranking of genes from lowest to highest CV out of 25,465 *M. guttatus* and 46,855 *M. l. luteus* genes. Only genes with mean expression greater than zero were included for ranking.

Expression variability (CV) was measured via RNA-seq across four tissue types for two biological replicates from each species. Genes are ordered from lowest to highest CV for each species. For *M. l. luteus,* all measures are the average of both homeologs.
