# Supplementary material for: A Whole-Transcriptome Approach to Evaluating Reference Genes for Quantitative Gene Expression Studies: A Case Study in Mimulus
Source: G3 (Bethesda). 2017 Mar 3;7(4):1085–95. doi: 10.1534/g3.116.038075 (PMC5386857; doi:10.1534/g3.116.038075)
Supplement: Supplementary file 6 [file 1085TableS3.docx]

**Table S3**. **Mean expression and expression variability of eight selected genes as measured by qPCR, T1 RNA-seq, and T2 RNA-seq.** See Figure 1 for a description of the plant materials that were sourced for each of these methods. Expression variability is measured in CV (where CV = SD/Mean) over the four tissue types (calyx, leaf, petal, stem). For the qPCR data, variability was calculated from mean expression, over the four biological replicates, of each tissue type; only one individual plant from each species was used for the T1 and T2 datasets.

| Type | Species | Gene^a^ | T1 Mean (FPKM) | T1 CV | T1 CV Rank^b^ | T2 Mean (FPKM) | T2 CV | T2 CV Rank^b^ | qPCR Mean^c^ | qPCR CV | qPCR CV Rank^d^ |
| --- | --- | --- | --- | --- | --- | --- | --- | --- | --- | --- | --- |
| Traditional | *M. guttatus* | ACT | 417.93 | 0.53 | 9,822 | 108.15 | 0.32 | 5,772 | 1.237 | 0.53 | 4 |
|  |  | GAP | 674.13 | 0.47 | 8,523 | 109.76 | 0.18 | 2,128 | 4.854 | 0.20 | 1 |
|  |  | PEX | 47.00 | 0.38 | 6,165 | 12.24 | 0.26 | 4,229 | 0.177 | 0.50 | 3 |
|  |  | UBC | 28.38 | 0.21 | 1,561 | 4.41 | 0.18 | 2,202 | 0.282 | 0.62 | 6 |
|  | *M. l. luteus* | ACT | 303.48 | 0.22 | 7,907 | 100.91 | 0.47 | 16,329 | 1.008 | 0.47 | 3 |
|  |  | GAP | 516.71 | 0.23 | 8,663 | 74.42 | 0.25 | 5,689 | 3.083 | 0.57 | 6 |
|  |  | PEX^e^ | 72.43 | 0.26 | 6,027 | 21.69 | 0.17 | 4,882 | 0.282 | 0.40 | 1 |
|  |  | UBC | 8.90 | 0.01 | 23 | 2.68 | 0.26 | 6,373 | 0.235 | 0.43 | 2 |
|  |  |  |  |  |  |  |  |  |  |  |  |
|  |  |  |  |  |  |  |  |  |  |  |  |
| Other | *M. guttatus* | MRP | 7.37 | 0.04 | 21 | 2.41 | 0.27 | 4,713 | 0.17 | 0.66 | 7 |
|  |  | PAE | 102.27 | 0.05 | 39 | 53.73 | 0.79 | 14,232 | 0.305 | 1.03 | 8 |
|  |  | RPK | 3.41 | 0.05 | 46 | 5.63 | 0.31 | 5,600 | 0.092 | 0.31 | 2 |
|  |  | ZNF | 11.61 | 0.02 | 4 | 6.83 | 0.36 | 7,002 | 0.158 | 0.60 | 5 |
|  | *M. l. luteus* | MRP | 4.87 | 0.07 | 1,162 | 0.87 | 0.20 | 3,568 | 0.021 | 0.55 | 5 |
|  |  | PAE | 60.29 | 0.10 | 1,934 | 23.26 | 0.56 | 20,058 | 0.228 | 0.70 | 8 |
|  |  | RPK^e^ | 17.77 | 0.01 | 48 | 10.01 | 0.55 | 17,575 | 0.084 | 0.69 | 7 |
|  |  | ZNF | 5.05 | 0.09 | 1,645 | 1.19 | 0.28 | 7,341 | 0.071 | 0.51 | 4 |

^a^ Gene acronyms: MRP = Mediator of RNA pol 12, PAE = pectin acetylesterase, RPK = receptor-like protein kinase, ZNF = FYVE zinc-finger transcription factor, ACT = actin 7, GAP = GAPDH-C1, PEX = peroxisome ubiquitin conjugating enzyme 4, UBC = ubiquiting conjugating enzyme. ^b^ Rank is determined based on lowest to highest CV of all genes with non-zero means, and is measured for each biological replicate separately. ^c^ Expression was relativized to an inter-plate calibrator and adjusted to PCR efficiency values. ^d^ Rank determined based on the lowest to highest CV of all eight traditional and novel genes. ^e^ Mean values for transcritome expression is the sum of the expression of two RPK homeologs and of three PEX homeologs; transcriptome CV is based on these summed expression values and CV rank is the average of homeolog ranks.

See Figure 1 for a description of the plant materials that were sourced for each of these methods. Expression variability is measured in CV (where CV = SD/Mean) over the four tissue types (calyx, leaf, petal, stem). For the qPCR data, variability was calculated from mean expression, over the four biological replicates, of each tissue type; only one individual plant from each species was used for the T1 and T2 datasets.
