# Supplementary material for: A Whole-Transcriptome Approach to Evaluating Reference Genes for Quantitative Gene Expression Studies: A Case Study in Mimulus
Source: G3 (Bethesda). 2017 Mar 3;7(4):1085–95. doi: 10.1534/g3.116.038075 (PMC5386857; doi:10.1534/g3.116.038075)
Supplement: Supplementary file 7 [file 1085TableS4.docx]

**Table S4.** **Forward and reverse pair primer pairs for four novel reference genes and four traditional reference genes.**

| Target Gene^a^ | Sequence^b^ | Distance from 3' (bp) | Product Size (bp) | Surrounds Intron? | Anneal Temp |
| --- | --- | --- | --- | --- | --- |
| ACT | GCCCCTCGTCTGTGATAATG | 1600 | 130 | Yes | 55C |
| Mgu_09880 | CTGTCCCATACCAACCATCA |  |  |  |  |
| ACT | ACTCTCTTCACCACCAGCTTTT | 1700 | 197 | Yes | 55C |
| Mlu_06653 | CTGTCCCATACCAACCATCA |  |  |  |  |
| GAP | TTGAAGGGAATCTTGGGCTA | 300 | 242 | Yes | 55C |
| Mgu_20644; Mlu_02705 | CATTTGACGTACCATAAACGAGTA |  |  |  |  |
| MRP | TATTCCCCAGTTCTGGATGG | 500 | 180 | No | 55C |
| Mgu_06967 | CGTGGGAGATCATAATCGAGA |  |  |  |  |
| MRP | AAGTCGTGATTTGGACAGGAA | 250 | 248 | No | 55C |
| Mlu_20604 | TTCTCGACAGTGCAGGAAAA |  |  |  |  |
| PAE | CATTGTGACGAGTTTCGTACTTT | 400 | 256 | Yes | 55C |
| Mgu_06532; Mlu_16546 | CCGCGTTGAGAAGAAAGAGA |  |  |  |  |
| PEX | TGGAATTTTCCAGCTTGCTT | 600 | 169 | Yes | 53C |
| Mgu_04762; Mlu_23196; Mlu_21920; Mlu_26129 | AACTGATTGCAGGGTCCAAG |  |  |  |  |
|  |  |  |  |  |  |
| RPK | CCTGAGGGTGACAAGACACA | 600 | 169 | Yes | 55C |
| Mgu_09659; Mlu_25834; Mlu_26332 | GCCTGCTTTTGTCCATTGAT |  |  |  |  |
|  |  |  |  |  |  |
| UBC | AGAAGGCCTCCAAAGTATT | 500 | 159 | Ex-Ex junction | 48C |
| Mgu_10589; Mlu_29747 | GCAGTTCTTATCTCCTTCGT |  |  |  |  |
| ZNF | TTGGAAAATGCCAAGATGC | 1300 | 194 | Ex-Ex junction | 55C |
| Mgu_04783 | CCCTCTGCGTCATTGAAACT |  |  |  |  |
| ZNF | TTACATGCCAATGCACTTCCAGTT | 600 | 189 | No | 55C |
| Mlu_26153 | CGTCGGGCAAAAGATAAAAA |  |  |  |  |
| RPK 5' | TGGGCTCGAGTATTTTGCTT | 1500 | 194 | No | 53C |
| Mgu_09659; Mlu_25834; Mlu_26332 | TGCTTCCTAATCCAAAGATACCA |  |  |  |  |
|  |  |  |  |  |  |

^a^ ACT = actin 7; GAP = GAPDH C2; MRP = Mediator of RNA polymerase II subunit 12; PAE = pectin acetylesterase; PEX = *PEX4*, a ubiquitin conjugating enzyme; RPK = receptor-like protein kinase; UBC = ubiquitin conjugating enzyme 26; ZNF = FYVE-type zinc finger transcription factor; Mgu = *M. guttatus*; Mlu = *M. l. luteus* ^b^ All primers listed in the 5’ to 3’ direction

Gene IDs refer to the unique ID given to RNAseq transcripts.
