# Supplementary material for: A Whole-Transcriptome Approach to Evaluating Reference Genes for Quantitative Gene Expression Studies: A Case Study in Mimulus
Source: G3 (Bethesda). 2017 Mar 3;7(4):1085–95. doi: 10.1534/g3.116.038075 (PMC5386857; doi:10.1534/g3.116.038075)
Supplement: Supplementary file 8 [file 1085TableS5.docx]

**Table S5**. **Reverse-transcription processivity based on the 3’/5’ amplification ratio of the receptor-like protein kinase gene.**

| Species | Tissue | Sample | 3'/5' Ratio^a^ |
| --- | --- | --- | --- |
| *M. guttatus* | Calyx | 1 | 5.077112957 |
|  |  | 2 | 6.487101437 |
|  |  | 3 | 6.690492088 |
|  |  | 4 | 4.57740583 |
|  | Leaf | 1 | 2.941528029 |
|  |  | 2 | 3.876806799 |
|  |  | 3 | 4.309049312 |
|  |  | 4 | 3.083876318 |
|  | Petal | 1 | 4.995999919 |
|  |  | 2 | 5.257557887 |
|  |  | 3 | 5.10192371 |
|  |  | 4 | 5.155170565 |
|  | Stem | 1 | 7.488874661 |
|  |  | 2 | 9.716297423 |
|  |  | 3 | 6.926328949 |
|  |  | 4 | 6.512940422 |
| *M. l .luteus* | Calyx | 1 | 1.277882998 |
|  |  | 2 | 0.661916579 |
|  |  | 3 | 0.770417742 |
|  |  | 4 | 0.93609711 |
|  | Leaf | 1 | 0.52385052 |
|  |  | 2 | 1.21520537 |
|  |  | 3 | 0.578333938 |
|  |  | 4 | 0.835695207 |
|  | Petal | 1 | 1.118039059 |
|  |  | 2 | 1.026442656 |
|  |  | 3 | 0.842665039 |
|  |  | 4 | 0.808940175 |
|  | Stem | 1 | 0.62998836 |
|  |  | 2 | 0.843927435 |
|  |  | 3 | 1.259371319 |
|  |  | 4 | 0.813809377 |

^a^ Expression values were normalized against an inter-plate calibrator and adjusted for PCR efficiency before the ratio was taken. The 5’ primer pair is 1000bp upstream of the 3’ primer pair.

The acceptable range of the 3’/5’ ratio is considered to be 0.2-5 by the MIQE guidelines (Bustin *et al.* 2009). Processivity was much lower for the *M. guttatus* samples, especially in the calyx and stem tissues. This could have an effect on genes with primers in the 5’ region of the gene, which includes ACT and ZNF.
