# Supplementary material for: A Whole-Transcriptome Approach to Evaluating Reference Genes for Quantitative Gene Expression Studies: A Case Study in Mimulus
Source: G3 (Bethesda). 2017 Mar 3;7(4):1085–95. doi: 10.1534/g3.116.038075 (PMC5386857; doi:10.1534/g3.116.038075)
Supplement: Supplementary file 9 [file 1085TableS6.docx]

**Table S6. Primer efficiency and R^2^ values for all qPCR runs.**

| Gene^a^ | Species^b^ | R^2^ | Efficiency (%)^c^ |
| --- | --- | --- | --- |
| ACT | MLL | 0.999 | 90.6 |
|  | MGUT | 0.999 | 86.6 |
| GAP | MLL | 0.998 | 91.4 |
|  | MGUT | 1 | 93.5 |
| MRP | MLL | 0.998 | 87.3 |
|  | MGUT | 0.988 | 93.4 |
| PAE | MLL | 0.999 | 86.1 |
|  | MGUT | 0.998 | 83.5 |
| PEX | MLL | 1 | 86.2 |
|  | MGUT | 0.992 | 93.8 |
| RPK | MLL | 0.997 | 94.5 |
|  | MGUT | 1 | 83.4 |
| UBC | MLL | 0.990 | 92.2 |
|  | MGUT | 0.998 | 92.5 |
| ZNF | MLL | 0.999 | 101.7 |
|  | MGUT | 0.999 | 86.2 |
| RPK 5' | MLL | 0.995 | 85.0 |
|  | MGUT | 0.999 | 94.4 |

^a^ ACT = actin 7; GAP = GAPDH C2; MRP = Mediator of RNA polymerase II subunit 12; PAE = pectin acetylesterase; PEX = *PEX4*, a ubiquitin conjugating enzyme; RPK = receptor-like protein kinase; UBC = ubiquitin conjugating enzyme 26; ZNF = FYVE-type zinc finger transcription factor ^b^ MLL = *M. l. luteus,* MGUT = *M. guttatus* ^c^ Efficiency calculated using 4-6 points from a ¼ dilution curve where E = 10^(1/-slope)^.
